# Resection of the Superior Maxilla for Sarcoma

**Published:** 1888-07

**Authors:** Herman Mynter

**Affiliations:** Surgeon to the Buffalo General Hospital


### RESECTION OF THE SUPERIOR MAXILLA FOR SARCOMA.

Read before a Union Meeting of the Seventh and Eighth District Dental Societies of the State of New York, held at Buffalo, October 25 and 26, 1887.

BY HERMAN MYNTER, M. D., SURGEON TO THE BUFFALO GENERAL HOSPITAL.

*Mr. President and Gentlemen :—*

I have asked the privilege of bringing a patient before your association to-night, because he suffered from a disease for which one would naturally, at the outset, consult members of your profession, and because the disease, if recognized early, offers rather a favorable prognosis, while if overlooked or neglected in its incipiency it will require a formidable operation, and even then, in a majority of cases, will relapse and progress to a fatal termination, with great sufferings.

The patient is a German, sixty-one years of age, who was admitted to the General Hospital, June 14th, 1887. He has a good family record, and has always enjoyed good health. Ten years ago he was much troubled with a bad tooth in the right upper maxilla. After a time it was extracted, but a piece of the root was left, which troubled him more or less. Ten months ago he first noticed a swelling in that locality, which steadily increased in size, but without being particularly painful. He consulted a dentist six weeks previous to his entrance into the hospital, who extracted a piece of a root, and after that the swelling grew rapidly. During the last weeks before entering the hospital, he had severe shooting pains and much tenderness of the growth. He lost his appetite, swallowed with difficulty, and was considerably emaciated. By local inspection, a large tumor was seen to occupy the whole posterior half of the right superior maxilla, extending from the first bicuspid backward. The whole superior maxilla was uniformly enlarged and protruding; all the molar teeth were lacking, and their place and the hard palate occupied by a large, soft, fringed and granulating tumor, as large as an egg, which filled the whole right side of the mouth, emitting a very bad odor. Backwards it was easily circumscribed with the finger, and neither the soft palate nor the anterior pillars of the pharynx seemed to be involved. No secondary affection of the glands of the neck was discovered.

June 15th—Operation under ether. Tracheotomy was first performed, and a large silver canula introduced, through which the narcosis was continued. A large sponge, attached to a strong silk thread, was then introduced into the pharynx, to prevent entrance of blood into the larynx. The whole right superior maxilla was then removed, after Ferguson's method. An incision was made from the inner angle of the eye to the outer limit of the orbit, parallel with and a little below the infraorbital ridge, as described in Agnew's Surgery, and another, commencing at the nasal extremity of the first, was carried down along the side of the nose around its ala to the columna nasi, and from there through the middle of the upper lip. The whole flap was then dissected up close to the bone, and the bleeding stopped by pressure with the fingers and a few ligatures. The periosteum at the floor of the orbit was then detached, a chain-saw introduced through the inferior fissura orbitalis and the malar bone, os zygoma, cut through. The processus frontalis of the superior maxilla was then severed with the chisel, and lastly the processus palatinus cut through with a straight narrow saw, introduced through the nose. The superior maxilla, thus loosened from its bony connections, was then seized with a strong pair of lion forceps, dislodged from its bed and drawn downwards, by which proceeding its attachment to the pterygoid process of the sphenoid was broken. Lastly, the soft palate was severed with the thermocautery. After the moderate hemorrhage was stopped, partly with ligatures, partly with the thermocautery, the wound was sutured with a great number of catgut sutures, and the large cavity filled with iodoform gauze.

June 16th—The patient has rallied well and takes nourishment. The tracheal canula was removed.

June 20th—The gauze packing was removed. Some sloughing of superficial tissue was observed, due to the thermocautery.

June 23d—The wound in the trachea is closed; the patient is sitting up, and the wound has united by first intention.

June 25th—Patient discharged, with the large cavity granulating and rapidly filling up.

By microscopical examination the tumor was found to be a round-celled sarcoma. Since the operation four months have passed, and no symptom of relapse has so far presented itself. We may therefore fairly consider him cured, although there is, of course, a possibility of future relapse. He feels perfectly well, is able to work, but swallows with some difficulty on account of the large opening in the hard palate, and his voice has a very pronounced nasal sound, so much so that it is difficult to understand him. For that reason, Dr. Barrett has made him a prothesis of hard rubber, attached to the teeth of the left superior maxilla, by the aid of which his speech, as you will observe, is very much improved and the swallowing easier. A cicatricial contraction of the mucous membrane, the result of the application of the thermocautery, by which he is prevented in opening his mouth widely, has given Dr. Barrett considerable difficulty in taking the impression. The contracted bands might, of course, be cut through without difficulty. The deformity, as you will observe, is very slight, on account of the dense connective tissue which fills out the void.

Our knowledge of tumors of the upper jaw has been modified by modern histological and microscopical investigations. I shall not here enter deeply into this subject, but only briefly state that tumors of the jaw may be either non-malignant, as fibromata, osteomata and enchondromata, or malignant, as sarcomata and carcinomata, with their subdivisions, scirrhous, medullary cancer and epithelioma. The non-malignant tumors take their origin either in the periosteum or the endosteum, may grow to an enormous size, but are not dangerous to life except by their size and complications arising from it; nor do they relapse after extirpation. Under the name sarcoma is understood tumors composed of tissue which is either embryonic, or which is undergoing one of the primary modifications seen in the development of adult connective tissue (Ericksen). We distinguish different forms of sarcoma as spindle-celled, round-celled, fibro-sarcoma, chondro-sarcoma, osteo-sarcoma, etc. We may, in general, say that the richer they are in cells, the more apt are they to relapse after extirpation ; the more connective tissue they contain, the less malignant. Free removal is necessary on account of their tendency to extend along the periosteum beyond the defined tumor.

The carcinomata are tumors of epithelial origin, and generally of alveolar structure. They infiltrate the affected part, producing induration and ulceration, with secondary affections of the lymphatic glands and the internal organs. Carcinomata generally attack people in middle life or old age, men twice as often as women. As regards the frequency of the different forms of tumors, no great reliance can be placed on the older statistics, gathered together before microscopical examinations of the extirpated tumors had become the rule. Dr. Ohleman (*Arkiv fuer Klinische Chirurgie,* 1875) reports thirty-two cases of total excision, of which nineteen were carcinomata, twelve sarcomata and one an echondroma. It shows the carcinomatous growths to be the most frequent, and in this agrees with other statistics. When we consider the question of the percentage of cures after the operation, there is more disagreement among the authors.

Ashhurst mentions eighty-four cases, of which fifty-one recovered and thirty-three died or relapsed, being sixty-one per cent. of recoveries. Agnew gives a table of one hundred and seventy-nine complete excisions, of which eighty recovered, being forty-five per cent. of recoveries.

Ohleman (*Archiv. fuer Klinische Chirurgie,* 1875) mentions thirty-two cases. Of these twenty were total excisions, with seventeen recoveries and three deaths, or eighty-five per cent. of recoveries, but relapse occurred in all cases, some earlier, some later (one to three years), and in no case was the cure permanent.

Braun (*Centrablatt fuer Chirurgie,* 1876) reports eleven cases, of which seven recovered and four died, but of the seven recoveries five had relapses, and died later, so that only two were permanently cured, being eighteen per cent.

Estlander (in *Nordisk Medicinsk Archiv.*) gives reliable but small statistics of twenty cases, which he followed to the end. Of these fifteen were men, five women, and the ages ranged from twenty-seven to sixty-nine. Operation was impossible in four, and four others died after the operation, of intercurrent diseases. Of the twelve left, two were finally cured, being seventeen per cent., while the rest died of relapse. You see, then, there is a great difference of opinion, some giving eighty-five per cent. of recoveries, others only seventeen per cent., yet the statistics from smaller countries are apt to be more reliable, as the cases generally may be followed to the end.

Estlander draws some conclusions from his statistics, which are interesting. The average duration is generally about one year, the progress being a little more rapid with younger patients than with older ones. The patients generally present themselves to the surgeon just midway between the first appearance of the tumor and the final fatal termination, so that the patient will live just about as long after the consultation as he has had the tumor before the consultation, and that, whether he be operated upon or not (cures not counted). Thus, if a patient has suffered six months before the consultation, he will probably live six months more; if he presents himself sooner (because the disease has made more rapid progress), he will die correspondingly sooner, but his sufferings will be mitigated and death made easier by the operation.

A patient with sarcoma would, according to this, ordinarily live one year. During the first six months the sufferings are not very acute, and he would therefore not apply for surgical aid. If not operated upon he would still have six months to live. The same would be the case if a relapse occurred after an operation. If he submits to an operation, his chances of a permanent cure are equal to one out of six. Secondarily, he has a chance of prolonging his life twice as long, but the probability that he will neither gain nor lose anything, as far as time is concerned, is four to one. He is only sure that the last months of his life will be more endurable if he be not cured. In partial excisions, the percentage of cures is much greater, while it is slightly worse in total excision of both superior maxillæ. The earlier the operation is performed, the greater is the probability of complete cure, and for that reason it is of the greatest importance to the patient that the disease be recognized early, when the tumor is yet small, and may require only a partial instead of a total excision. In regard to etiology, very little is known. Dr. Ohleman states that in some cases heredity was evident, in others an injury. In most cases no cause can be given, and it is impossible to decide whether a trauma gives the occasion to the abnormal development of the cells, or only favors it by the irritation it produces. The pain is generally ascribed to the teeth, and as they become loose they are extracted. In the course of six months the patients generally present themselves with a tumor as big as an egg, but otherwise in good condition, and without secondary affections of the glands. The differential diagnosis is not difficult. The slow and painless development of the tumor, its appearance, the uniform enlargement, the loosening of the teeth and the extension along the gum, are sufficient to distinguish the trouble from periostitis, with its severe pain, increased generally by pressure on one tooth, its inflammatory symptoms and its hard, painful swelling of the bone itself. Abscess of the Antrum of Highmore presents more symptoms of inflammation, the etiology is better known and the dry parchment sensation is characteristic. An exploratory puncture will reveal pus. Lastly, a microscopical examination of a small piece of the tumor will guard against mistakes.

A few points deserve mention in regard to the operation itself. The greatest danger is the hemorrhage, and the entrance of blood into the larynx and lungs. That the patients in some measure might voluntarily expectorate the blood, the operation was formerly often performed without narcosis, or with only partial narcosis during the beginning of the operation. Even if this in some measure relieves the danger of entrance of blood into the larynx, it lengthens the operation considerably by the violent struggles of the patient, and the whole performance is horrible beyond expression. We have two other ways of overcoming the difficulty, either preliminary tracheotomy, as in my case, or Roser's method of operating with declined head. Roser's method is liable to the objection that it increases the bleeding and is apt to produce acute anæmia. Max Müller mentions such a case (*Arkiv. fuer Klinische Chirurgie,* 1875), and states that he never saw such a bleeding in twelve previous cases in which Roser's method was not used. By preliminary tracheotomy we avoid this danger, and the narcosis can with the greatest ease be maintained through the tube. A Trendelenburg's canula is not necessary at all, as the pharynx can be blocked with a sponge to which a string is attached, that the sponge may not be swallowed, and not a drop of blood can enter the larynx. But tracheotomy has one disadvantage; it produces paresis of the vocal chords, and therefore it may be necessary to feed the patient for some days, either per rectum or with a stomach tube, to prevent the food from entering the lungs, which may be followed by pneumonia.

I will mention one improvement in the operation, described by Létiévant. He conserves the infraorbital nerve, and by that the contractility of the muscles of the face, he says, by chiseling open the canal and taking the nerve out. The statement about conserving the contractility of the muscles of the face is, of course, a mistake, as the infraorbital nerve is not a motor nerve. Yet his operation has the advantage that it leaves on both sides of the nerve a part of the bony margin of the orbit, and a part of the alveolar process with the incisor teeth. These three points prevent the sinking in of the flap and give it a good support.

---

At the present moment, one year after the operation, the patient is enjoying excellent health, and absolutely no signs of relapse have occurred. The cavity has still further contracted, and, the nasal tone excepted, gives him no trouble. The cicatricial contraction of the mucous membrane has increased some, and prevents him in opening the mouth far enough to have the prothesis inserted. It would be quite difficult to overcome this contraction now. Extirpation of the contracted bands would have no effect, as the muscles probably are very much shortened too, but there is really no indication for interfering with it, as he is able to open the mouth sufficient to masticate his food.

